# Comparative Study of Circulating MMP-7, CCL18, KL-6, SP-A, and SP-D as Disease Markers of Idiopathic Pulmonary Fibrosis

**DOI:** 10.1155/2016/4759040

**Published:** 2016-05-17

**Authors:** Kosuke Hamai, Hiroshi Iwamoto, Nobuhisa Ishikawa, Yasushi Horimasu, Takeshi Masuda, Shintaro Miyamoto, Taku Nakashima, Shinichiro Ohshimo, Kazunori Fujitaka, Hironobu Hamada, Noboru Hattori, Nobuoki Kohno

**Affiliations:** ^1^Department of Molecular and Internal Medicine, Graduate School of Biomedical and Health Sciences, Hiroshima University, 1-2-3 Kasumi, Minami-ku, Hiroshima 734-8551, Japan; ^2^Department of Respiratory Medicine, Hiroshima Prefectural Hospital, 1-5-54, Ujina-Kanda, Minami-ku, Hiroshima 734-0004, Japan; ^3^Department of Physical Analysis and Therapeutic Sciences, Graduate School of Biomedical and Health Sciences, Hiroshima University, Hiroshima 734-8551, Japan

## Abstract

*Background.* Recent reports indicate that matrix metalloproteinase-7 (MMP-7) and CC-chemokine ligand 18 (CCL18) are potential disease markers of idiopathic pulmonary fibrosis (IPF). The objective of this study was to perform direct comparisons of these two biomarkers with three well-investigated serum markers of IPF, Krebs von den Lungen-6 (KL-6), surfactant protein-A (SP-A), and SP-D.* Methods.* The serum levels of MMP-7, CCL18, KL-6, SP-A, and SP-D were evaluated in 65 patients with IPF, 31 patients with bacterial pneumonia, and 101 healthy controls. The prognostic performance of these five biomarkers was evaluated in patients with IPF.* Results.* The serum levels of MMP-7, KL-6, and SP-D in patients with IPF were significantly elevated compared to those in patients with bacterial pneumonia and in the healthy controls. Multivariate survival analysis showed that serum MMP-7 and KL-6 levels were independent predictors in IPF patients. Moreover, elevated levels of both KL-6 and MMP-7 were associated with poorer survival rates in IPF patients, and the combination of both markers provided the best risk discrimination using the C statistic.* Conclusions.* The present results indicated that MMP-7 and KL-6 were promising prognostic markers of IPF, and the combination of the two markers might improve survival prediction in patients with IPF.

## 1. Introduction

Idiopathic pulmonary fibrosis (IPF) is a progressive fibrotic lung disease of unknown etiology with a median survival of 2-3 years from the time of diagnosis. At present, high-resolution computed tomography (HRCT) is an essential component of the diagnosis of IPF [1]. Surgical lung biopsy and bronchoscopic examination also have an important role in the diagnosis of IPF. Serial lung function testing is generally used to monitor disease progression and to predict prognosis [2, 3], but the clinical course of IPF is highly variable and unpredictable. Therefore, noninvasive blood biomarkers with diagnostic and prognostic utility could support the diagnosis of IPF, especially in settings with limited medical resources, and would help in the identification of vulnerable patients.

Krebs von den Lungen-6 (KL-6), surfactant protein-A (SP-A), and SP-D are type II pneumocyte-derived molecules which have been investigated by our group and other investigators for their usefulness as serum biomarkers of IPF [4–6]. These molecules are being widely used in clinical practice in Japan as serum markers of interstitial lung diseases (ILDs). Serum levels of these markers increase in patients with IPF, and high serum levels of these markers were shown to be associated with poorer survival in IPF [7, 8]. We have previously conducted a comparative study of these three markers and demonstrated that KL-6 had the best diagnostic value for differentiating 33 patients with ILDs from 82 control subjects, that is, healthy volunteers and patients with bacterial pneumonia [9].

In addition to the abovementioned type II pneumocyte-derived biomarkers, recent reports indicate that matrix metalloproteinase-7 (MMP-7) and CC-chemokine ligand 18 (CCL18) are potential diagnostic and prognostic markers of IPF. MMP-7 has been shown to be upregulated in the lungs in IPF, particularly in alveolar macrophages and hyperplastic epithelial cells [10]. Bronchoalveolar lavage fluid (BALF) and serum levels of MMP-7 are significantly higher in patients with IPF compared with those in healthy subjects [11]. Elevated levels of serum MMP-7 are associated with impaired lung function and poorer survival in IPF patients [11, 12]. CCL18, a CC-chemokine produced by human myeloid cells, is abundantly secreted by alveolar macrophages in IPF patients. Previous reports demonstrated that serum levels of CCL18 were elevated in IPF patients, and elevated CCL18 levels were associated with poorer prognosis [13]. These results indicate that MMP-7 and CCL18 are candidate serum biomarkers of IPF; however, no previous investigations have compared the diagnostic and prognostic value of these two molecules with that of previously reported pneumocyte-derived biomarkers.

The aim of this study was to perform direct comparisons of the abovementioned five serum biomarkers as disease markers for IPF. We evaluated the serum levels of the five biomarkers in patients with IPF and control subjects, which consisted of patients with bacterial pneumonia (BP) and healthy controls (HC), and determined the relative values of these biomarkers in discriminating IPF patients from control subjects. Moreover, we examined independent predictive values of serum markers for survival of patients with IPF and tested their additive predictive ability compared with clinical information using the C statistic.

## 2. Materials and Methods

### 2.1. Subjects

Sixty-five patients with IPF, 31 patients with BP, and 101 HC were included in the present study. IPF was diagnosed by clinical features, laboratory findings, chest HRCT, and/or surgical lung biopsy, according to the ATS/ERS/JRS/ALAT statement [1]. Eleven patients with IPF underwent lung biopsy and were histologically diagnosed as usual interstitial pneumonia. The diagnosis of BP was based on infiltrative shadows on the chest X-ray and clinical symptoms. The HC were recruited from participants who underwent a health checkup, including a pulmonary function test and a chest X-ray, and those with malignancy or apparent lung disease were excluded. The survival analyses were performed in 62 patients with IPF, whose followup data were available for at least 6 months. This study was approved by the Ethics Committee of Hiroshima University, and written informed consent was obtained from all subjects.

### 2.2. Pulmonary Function Tests

Spirometric measurements, including vital capacity (VC) and forced expiratory volume in 1 second (FEV_1_), were performed according to the ATS/ERS recommendation [14]. Diffusing capacity of the lung for carbon monoxide (D_LCO_) was measured by the single-breath method in IPF patients, and lung function measurements were performed at diagnosis in these patients. Reference values were obtained from Japanese reference values for spirometry and D_LCO_, and the percentages of predicted normal values were calculated.

### 2.3. Serum Measurements

Blood samples were taken at diagnosis and stored at –80°C until analysis. MMP-7, CCL18, SP-A, and SP-D were measured by commercially available enzyme-linked immunosorbent assay (ELISA) kits (Human Total MMP-7 Quantikine ELISA Kit, R&D Systems, MN; Human CCL18/PARC Quantikine ELISA Kit, R&D Systems, MN; SP-A Test Kokusai-F Kit, Sysmex, Japan; and SP-D EIA Kit Yamasa, Yamasa, Japan). Serum KL-6 levels were measured by sandwich-type electrochemiluminescence immunoassay (ECLIA) using a Picolumi 8220 Analyzer (Eidia, Tokyo, Japan), as previously described [9].

### 2.4. Statistical Analysis

The results were expressed as the mean ± SD. Demographic characteristics and the levels of serum biomarkers were compared between the subject groups using Bonferroni's test. The levels of serum biomarkers were further analyzed by receiver operating characteristic (ROC) curves to determine the cut-off levels that resulted in the optimal diagnostic accuracy for each marker between the 65 patients with IPF and the 132 control subjects, including BP and HC. The use of these cut-off levels allowed the calculation of sensitivity, specificity, diagnostic accuracy, and likelihood ratio of the five biomarkers for separating the IPF patients from the control subjects. A likelihood ratio above 10 indicates strong diagnostic evidence [15].

In the survival analysis of the IPF patients, another ROC curve analysis was conducted to find an optimal cut-off level for the prediction of 5-year survival. The 5-year mortality between two groups was compared using the Kaplan-Meier method and the log rank test. Univariate and multivariate Cox proportional hazards model was used to identify predictors of 5-year survival in IPF patients. Martingale residuals plots were employed to check for assumptions of the proportional hazards and the linearity of each biomarker. The plots were visually evaluated with the help of locally weighted regression scatterplot smoothing [16]. Subsequently, the C statistic was evaluated to determine whether independent predictors of the multivariate analysis improved the discrimination for mortality of IPF patients when added to a baseline model as our previous report [17]. The C index is similar in concept to the area under the time-dependent ROC curve constructed by a Cox proportional hazards model [18]. A C index value between 0.70 and 0.80 is typically considered acceptable, whereas a value exceeding 0.80 is considered excellent [19]. Statistical analyses were done using the statistical software R version 3.2.2. The ROC curves were drawn with the “pROC” package, and the log rank test and Cox proportional hazards model were performed using the “survival” package of the R software. The C statistic was calculated using SPSS version 13.0 for Windows (SPSS Inc., Chicago, IL). Differences were considered statistically significant when the *p* value was < 0.05.

## 3. Results

### 3.1. Subject Characteristics

The mean age of the IPF patients was 69.3 years, and the patients with IPF were significantly older than the HC. The mean pack-years of smoking were 37.6 in IPF patients, which was significantly higher than those in the HC. There was no significant difference in age and smoking pack-years between the patients with IPF and those with BP. In the lung function analysis, the mean % VC in patients with IPF was significantly lower than that in the HC (Table 1).

### 3.2. Serum Concentrations of MMP-7, CCL18, KL-6, SP-D, and SP-A

**Figure 1:**
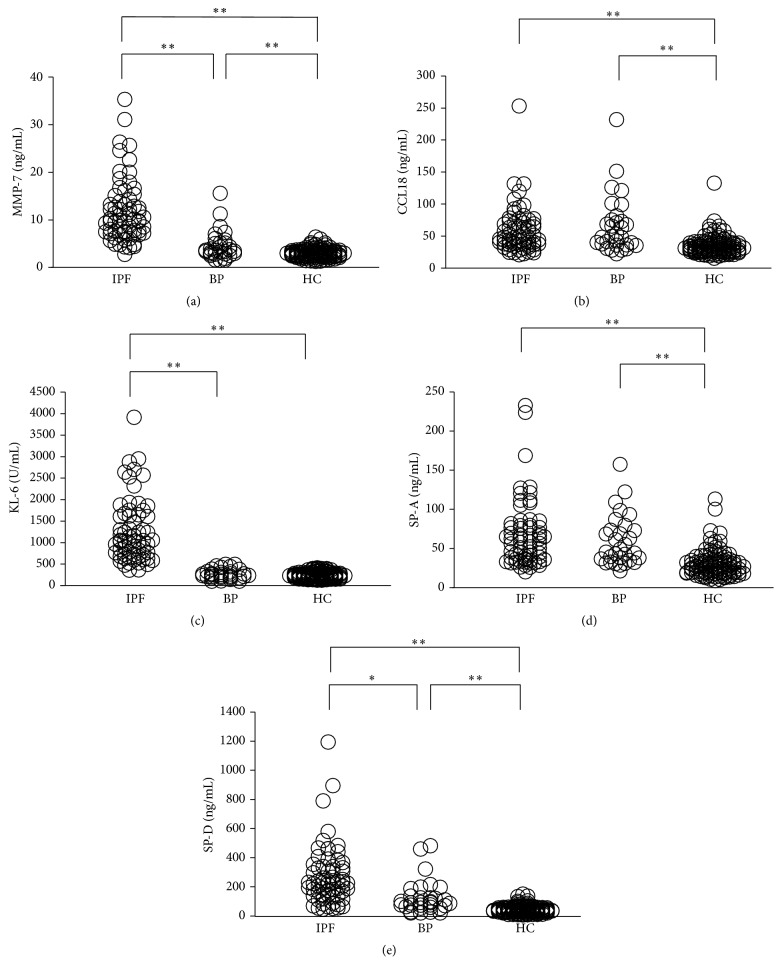
Serum levels of (a) MMP-7, (b) CCL18, (c) KL-6, (d) SP-A, and (e) SP-D in patients with IPF, those with bacterial pneumonia (BP), and healthy controls (HC). The significant differences between the three groups were evaluated using Bonferroni's test (^*∗*^ *p* < 0.01 and ^*∗∗*^ *p* < 0.001).

Baseline serum levels of the five biomarkers in patients with IPF were significantly higher than those in the HC. Moreover, serum levels of MMP-7, KL-6, and SP-D in patients with IPF were significantly elevated compared with those in patients with BP. However, there was no significant difference in the serum levels of CCL18 and SP-A between patients with IPF and patients with BP. Moreover, serum levels of MMP-7, CCL18, SP-A, and SP-D were significantly elevated in patients with BP compared with the HC (Figure 1).

### 3.3. ROC Curve Analysis for Discriminating IPF Patients from Control Subjects

**Figure 2:**
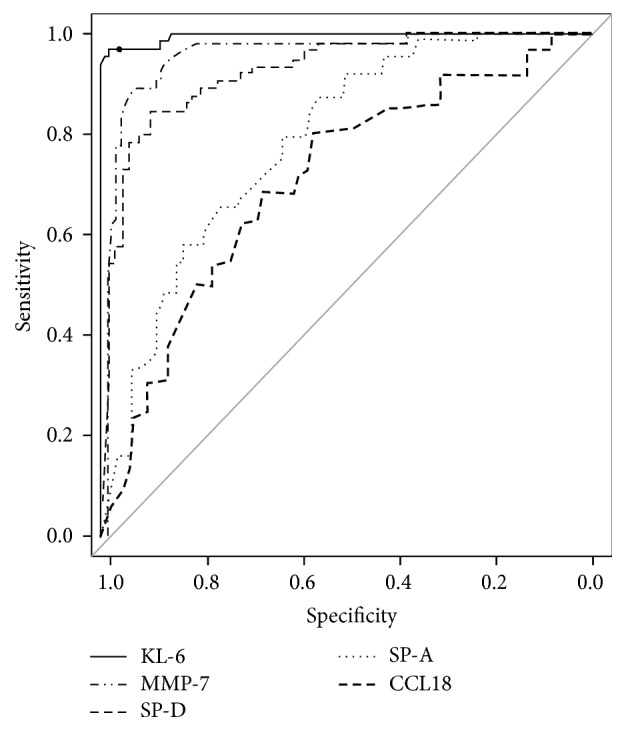
ROC curve analysis in five biomarkers to distinguish IPF patients from control subjects which consisted of patients with bacterial pneumonia and healthy controls.

ROC curve analysis was used to evaluate the discriminating capability of the five serum biomarkers to differentiate IPF patients from control subjects (Figure 2). Cut-off values were set as the levels that resulted in the optimal diagnostic accuracy for each marker: 5.56 ng/mL for MMP-7, 38.7 ng/mL for CCL18, 476 U/mL for KL-6, 44.0 ng/mL for SP-A, and 107.0 ng/mL for SP-D. The analysis of these levels indicated that KL-6 had the highest diagnostic accuracy (98.0%) and likelihood ratio (64.0). MMP-7 also showed a high diagnostic accuracy (91.4%) and likelihood ratio (12.9) (Table 2). We constructed another ROC curve using a logistic regression model including KL-6 and MMP-7. There was no significant difference between the combination of KL-6 and MMP-7 and either marker alone in the ability to discriminate between IPF and control subjects (Figure S1 in Supplementary Material available online at http://dx.doi.org/10.1155/2016/4759040). Subsequently, we evaluated the discriminative power of each biomarker to differentiate between IPF and BP and between IPF and HC, separately. Only KL-6 showed a high discriminatory ability whereas SP-A and CCL18 were poor indicators for discriminating IPF from BP (Table S1(a) and Figure S2(a)). On the other hand, all five biomarkers were useful to distinguish patients with IPF from the HC (Table S1(b) and Figure S2(b)).

### 3.4. Prognostic Values of Serum Biomarkers in IPF Patients

The median followup period in IPF was 31.0 (95% confidence interval: 26.6 to 35.4) months. To find an optimal cut-off level that could discriminate survivors from nonsurvivors, another ROC curve was drawn (figure not shown). Survival in IPF patients using biomarker levels above or below the cut-off level was estimated using the Kaplan-Meier method. Survival was significantly different between higher and lower levels of MMP-7, CCL18, and KL-6 (Figure S3).

In the univariate Cox analysis, decreased % VC, use of immunosuppressant drugs, and elevated serum levels of MMP-7 and KL-6 were associated with poor survival. In the multivariate analysis, only MMP-7 (hazard ratio (HR), 1.074; *p* = 0.0336) and KL-6 (HR, 1.001; *p* = 0.0042) were shown to be independent predictors for 5-year mortality (Table 3). The fit of the proportional hazard model was assessed by examining martingale residuals (Figure S4).

**Figure 3:**
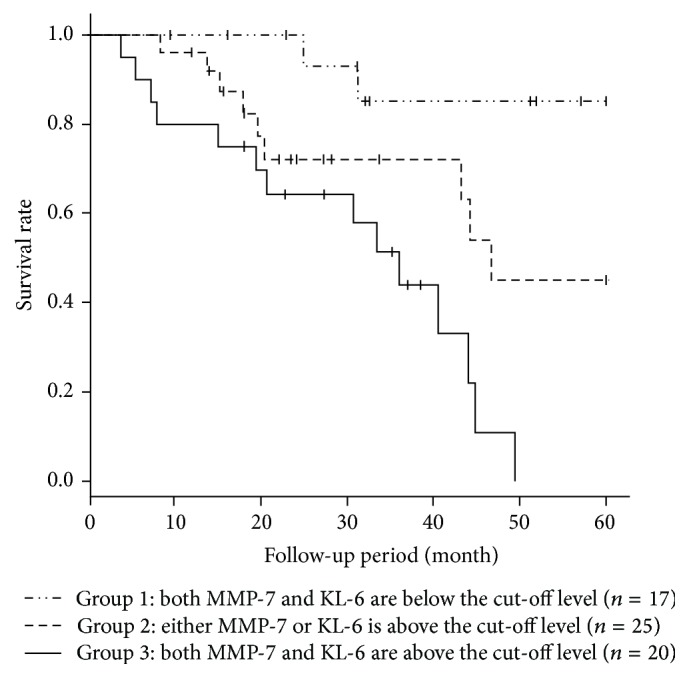
Kaplan-Meier analysis to evaluate the probability of 5-year survival among the three groups which were divided according to the serum levels of KL-6 and MMP-7. The cut-off levels of KL-6 and MMP-7 were 1040 U/mL and 9.67 ng/mL, respectively. The probability of 5-year survival was significantly different among them (*p* = 0.0004).

As shown in Figure 3, elevated levels of both KL-6 and MMP-7 were associated with poorer survival rates in IPF patients. The C statistic was used to determine whether the addition of biomarkers to the clinical model improved its predictive power. The C index for predicting mortality was 0.705 when clinical covariates (age, sex, and % VC) were included. The C index increased when MMP-7 and KL-6 were separately incorporated into a model with covariates (C index of 0.741 and 0.769, resp.). When the combination of MMP-7 and KL-6 was incorporated with covariates, the highest C index was obtained (0.816) (Table 4).

## 4. Discussion

In the present study, we directly compared the diagnostic and prognostic value of five serum biomarkers—MMP-7, CCL18, KL-6, SP-A, and SP-D—in patients with IPF and control subjects. Multivariate Cox analysis showed that serum levels of MMP-7 and KL-6 were independent predictors of prognosis in IPF patients. In addition, IPF patients with elevated levels of both KL-6 and MMP-7 had worse survival rates, and the combination of the two markers with the baseline covariates provided the highest C index. These findings indicated that both MMP-7 and KL-6 were promising prognostic markers of IPF, and a combination of the two markers might improve the survival prediction in patients with IPF. Additionally, we showed that MMP-7 and KL-6 could clearly differentiate IPF patients from patients with bacterial pneumonia and healthy controls, suggesting their potential as diagnostic biomarkers.

In this study, MMP-7 and KL-6 were independent predictors of prognosis in patients with IPF, which was consistent with the results of previous reports [7, 20, 21]. Moreover, the present results showed that IPF patients with elevated levels of both MMP-7 and KL-6 had poorer survival rates, suggesting that an assessment of both MMP-7 and KL-6 is more effective at identifying a high-risk subgroup than individual assessments of either biomarker. The results of the C statistic analysis showed that the combination of these two biomarkers with baseline risk factors might produce a more powerful prognostic model than either marker alone. MMP-7, a family of zinc-containing enzymes with proteolytic activity, and KL-6, a high molecular weight glycoprotein classified as a MUC1 mucin, have been suggested to be involved in the progression of IPF by different mechanisms. We have previously shown that KL-6 has chemotactic and antiapoptotic effects on fibroblasts in vitro [22, 23], indicating its putative role in the progression of fibrotic changes in the lung. MMP-7 is involved in extracellular matrix degradation and could also exert profibrotic effects by processing bioactive substrates, including heparin-binding EGF-like growth factor, insulin growth factor binding protein-3, and plasminogen [24]. The present results indicated that the combination of these two biomarkers might enable more accurate prediction of prognosis, but further prospective studies are needed to confirm this finding.

The present results indicate that MMP-7 could be a promising diagnostic marker of IPF. The ROC curve analysis showed the excellent discriminative capability of MMP-7, as indicated by the high area under the curve (AUC) value (>0.90) and likelihood ratio (>10). It should be noted that the ability of a biomarker to discriminate IPF from BP does not necessarily indicate that the biomarker is sufficient, by itself, for diagnosing IPF, although low false positive rates in BP would be an important feature for diagnostic markers for ILDs as shown in our previous study [9]. MMP-7 is expressed by airway epithelial cells and macrophages in impaired lungs in IPF, but not in normal lungs [10, 11]. Moreover, MMP-7 knockout mice are protected from bleomycin-induced lung fibrosis, indicating that MMP-7 actively participates in the tissue fibrotic response [25]. On the other hand, MMP-7 expression in the lungs can be upregulated by pneumococcal infection [26], which indicates the pivotal role of MMP-7 in the pathophysiology of lung infection. However, no published reports have determined circulatory levels of MMP-7 in patients with BP. This study demonstrated that although serum levels of MMP-7 were significantly elevated in patients with BP compared with the HC, serum MMP-7 levels were further elevated in IPF patients and could discriminate between IPF and BP.

Our study also demonstrated that serum levels of CCL18 had moderate discriminatory ability for differentiating IPF patients from the HC; however, CCL18 was found to be a poor indicator for distinguishing IPF from BP. An in vitro study reported that CCL18 had a chemotactic effect on lung fibroblasts and stimulated collagen production [27]. However, an in vivo study reported that the overexpression of CCL18 in mice enhanced bleomycin-induced lymphocytic inflammation but, paradoxically, attenuated collagen accumulation in the lungs [28], suggesting that complex mechanisms exist for the associations between CCL18 and fibrotic changes in the lung. With regard to CCL18 and bacterial infection, CCL18 was induced in peripheral blood mononuclear cells by staphylococcal enterotoxins and in alveolar macrophages by lipopolysaccharide and tuberculous infection [29, 30]. However, no previous reports have determined the serum levels of CCL18 in patients with BP. In this respect, the present study showed that serum levels of CCL18 in patients with BP were significantly elevated compared to those in the HC, and CCL18 was not discriminative between IPF and BP.

The present study had several limitations. First, this study is a retrospective review of patients with IPF prospectively recruited from one tertiary hospital, and only Japanese participants who agreed to join this study were included. Therefore, our results may not be generalized to all patients with IPF. Second, there are distinctions in age and smoking histories between patients with IPF and HC. Third, control groups in this study consisted of only patients with BP, an acute lung disease, and healthy subjects; patients with chronic lung diseases, especially other ILDs, were not included in the control group. Therefore, we did not fully evaluate the diagnostic utility of the serum biomarkers. It should be noted that no clinically useful biomarker for distinguishing IPF from other ILDs has been found. We have previously reported that serum levels of KL-6 were also elevated in patients with hypersensitivity pneumonia (HP) [4]. Ishii et al. reported that serum levels of SP-A, but not those of SP-D and KL-6, were significantly elevated in patients with IPF compared to patients with nonspecific interstitial pneumonia (NSIP) [31]. Additionally, serum levels of CCL18 were shown to be elevated in patients with HP when compared with patients with IPF and NSIP [32]. Therefore, future studies are needed to clarify whether biomarker panels can differentiate IPF from other ILDs.

## 5. Conclusions

Our results showed that both MMP-7 and KL-6 might be a useful prognostic marker of IPF, and a combination of the two markers may improve survival prediction in patients with IPF. Additionally, we showed that MMP-7 and KL-6 could differentiate IPF patients from patients with bacterial pneumonia and healthy controls. These results indicate that the measurement of serum levels of KL-6 and/or MMP-7 could potentially support the diagnosis of IPF and would be useful for identifying vulnerable patients especially when the two markers are used in combination. Further large-scale investigation would be warranted to confirm this finding and to find the best method to use this combination of biomarkers of IPF.

## Supplementary Material

Supplementary table 1 and supplementary figure 2 shows the diagnostic value and cut-off levels of the five biomarkers to discriminate patients with IPF from those with bacterial pneumonia and healthy subjects, separately.
Supplementary figure 1 shows the ROC curve analysis in serum levels of KL-6, MMP-7 and the combination of serum levels of KL-6 and MMP-7 to distinguish IPF patients from patients with bacterial pneumonia and healthy controls.
Supplementary figure 3 shows the Kaplan-Meier analysis of the IPF patients using biomarker levels.
Supplementary figure 4 shows the Martingale residuals plots employed to check for assumptions of the Cox proportional hazards model.

## Figures and Tables

**Table 1:** Subject characteristics.

	IPF	BP	HC
Subjects (*n*)	65	31	101
Age, yr	69.3 ± 8.5^*∗∗*^	67.8 ± 15.0^*∗∗*^	55.9 ± 2.3
Sex, M/F	50/15	21/10	76/25
Pack-years	37.6 ± 35.4^*∗*^	21.5 ± 26.7	13.7 ± 21.0
Spirometry			
% VC, %	74.5 ± 21.2^*∗∗*^	—	109.5 ± 13.2
FEV_1_/FVC, %	83.5 ± 17.0	—	80.6 ± 4.9
% D_LCO_, %	47.1 ± 15.8	—	—

IPF: idiopathic pulmonary fibrosis, BP: bacterial pneumonia, HC: healthy controls, VC: vital capacity, FEV_1_: forced expiratory volume in 1 second, FVC: forced vital capacity, and D_LCO_: diffusing capacity of the lung for carbon monoxide.

Data represent the mean ± SD.

Significant differences versus the HC were evaluated using Mann-Whitney *U* test.

^*∗*^ *p* < 0.001 and ^*∗∗*^ *p* < 0.0001.

**Table 2:** Cut-off values and the discriminatory ability of five biomarkers by ROC curve analysis, which distinguishes IPF patients (*n* = 65) from control subjects^*∗*^ (*n* = 132).

	MMP-7	CCL18	KL-6	SP-A	SP-D
AUC	0.9638	0.7036	0.9957	0.7865	0.9242
95% CI	0.9374–0.9901	0.6275–0.7815	0.9898–1.0020	0.7229–0.8501	0.8866–0.9619

Cut-off value	5.56 ng/mL	38.7 ng/mL	476 U/mL	44.0 ng/mL	107.0 ng/mL
Sensitivity	87.7%	66.2%	96.9%	66.2%	84.6%
Specificity	93.2%	67.4%	98.5%	76.5%	88.6%
Diagnostic accuracy	91.4%	67.0%	98.0%	73.1%	87.3%
Likelihood ratio	12.9	2.0	64.0	2.8	7.5

ROC: receiver operating characteristic, IPF: idiopathic pulmonary fibrosis, MMP-7: matrix metalloproteinase-7, CCL18: CC-chemokine ligand 18, KL-6: Krebs von den Lungen-6, SP-A: surfactant protein-A, SP-D: surfactant protein-D, AUC: area under the curve, and 95% CI: 95% confidence interval.

^*∗*^Control subjects consisted of 31 patients with bacterial pneumonia and 101 healthy controls.

**Table 3:** Cox proportional hazards model to predict the 5-year mortality of patients with IPF.

Variables	HR	95% CI	*p* value
Univariate analysis			
MMP-7 (continuous)	1.068	1.015–1.124	0.0109
CCL18 (continuous)	1.007	0.999–1.014	0.0734
KL-6 (continuous)	1.001	1.000–1.001	0.0005
SP-A (continuous)	1.006	0.999–1.015	0.1143
SP-D (continuous)	1.000	0.998–1.002	0.9180
Age	1.032	0.982–1.085	0.2128
Sex, M	2.163	0.734–6.370	0.1616
Smoking	1.468	0.546–3.951	0.4471
% VC (continuous)	0.965	0.942–0.989	0.0040
Medication^*∗*^	2.730	1.177–6.333	0.0193

Multivariate analysis^*∗∗*^			
MMP-7 (continuous)	1.074	1.060–1.147	0.0336
KL-6 (continuous)	1.001	1.000–1.002	0.0042
% VC (continuous)	0.981	0.954–1.009	0.1744
Medication^*∗*^	2.066	0.667–6.399	0.2086

See legends of Tables 1 and 2 for expansion of abbreviations.

^*∗*^Medication indicates the usage of corticosteroids and/or immunosuppressants.

^*∗∗*^Multivariate Cox analysis was adjusted for age, sex, and smoking history.

**Table 4:** C statistic for Cox regression models predicting 5-year mortality of patients with idiopathic pulmonary fibrosis.

	C index	95% CI
Covariates^*∗*^ only	0.705	0.559–0.851
Covariates plus MMP-7 (continuous)	0.741	0.605–0.876
Covariates plus KL-6 (continuous)	0.769	0.643–0.895
Covariates plus MMP-7 + KL-6 (continuous)	0.816	0.707–0.923

See legends of Tables 1 and 2 for expansion of abbreviations.

^*∗*^Covariates include age (continuous variable), sex, and percent predicted vital capacity.

## Abbreviations

AUC: Area under the curve
BALF: Bronchoalveolar lavage fluid
BP: Bacterial pneumonia
CCL18: CC-chemokine ligand 18
D_LCO_: Diffusing capacity of the lung for carbon monoxide
ECLIA: Electrochemiluminescence immunoassay
ELISA: Enzyme-linked immunosorbent assay
FEV_1_: Forced expiratory volume in 1 second
HC: Healthy controls
HP: Hypersensitivity pneumonia
HR: Hazard ratio
HRCT: High-resolution computed tomography
ILDs: Interstitial lung diseases
IPF: Idiopathic pulmonary fibrosis
KL-6: Krebs von den Lungen-6
MMP-7: Matrix metalloproteinase-7
NSIP: Nonspecific interstitial pneumonia
ROC: Receiver operating characteristic
SP-A: Surfactant protein-A
SP-D: Surfactant protein-D
VC: Vital capacity.
