# Supplementary material for: Comparative Study of Circulating MMP-7, CCL18, KL-6, SP-A, and SP-D as Disease Markers of Idiopathic Pulmonary Fibrosis
Source: Dis Markers. 2016 May 17;2016:4759040. doi: 10.1155/2016/4759040 (PMC4886062; doi:10.1155/2016/4759040)
Supplement: Supplementary file 1 — Supplementary table 1 and supplementary figure 2 shows the diagnostic value and cut-off levels of the five biomarkers to discriminate patients with IPF from those with bacterial pneumonia and healthy subjects, separately. Supplementary figure 1 shows the ROC curve analysis in serum levels of KL-6, MMP-7 and the combination of serum levels of KL-6 and MMP-7 to distinguish IPF patients from patients with bacterial pneumonia and healthy controls. Supplementary figure 3 shows the Kaplan-Meier analysis of the IPF patients using biomarker levels. Supplementary figure 4 shows the Martingale residuals plots employed to check for assumptions of the Cox proportional hazards model. [file 4759040.f1.pdf]

Supplementary materials

Comparative Study of Circulating MMP-7, CCL18, KL-6, SP-A, and SP-D as Disease Markers of Idiopathic Pulmonary Fibrosis

Authors

Kosuke Hamai[1], Hiroshi Iwamoto[1]*, Nobuhisa Ishikawa[2], Yasushi Horimasu[1], Takeshi Masuda[1], Shintaro Miyamoto[1], Taku Nakashima[1], Shinichiro Ohshimo[1], Kazunori Fujitaka[1], Hironobu Hamada[3], Noboru Hattori[1], Nobuoki Kohno[1]

[1] Department of Molecular and Internal Medicine, Graduate School of Biomedical and Health Sciences, Hiroshima University

[2] Department of Respiratory Medicine, Hiroshima Prefectural Hospital

[3] Department of Physical Analysis and Therapeutic Sciences, Graduate School of Biomedical and Health Sciences, Hiroshima University

***Corresponding author**

Hiroshi Iwamoto, MD, Ph.D.

TABLE S1: Cut off values and the discriminatory ability of five biomarkers by ROC curve analysis, which distinguishes patients with IPF from (a) BP and (b) HC.

(a)

	MMP-7	CCL18	KL-6	SP-A	SP-D
AUC	0.8998	0.5365	0.9918	0.5494	0.7866
95% CI	0.8280–0.9715	0.6642–0.4088	0.9806–1.0030	0.4250–-0.6738	0.6852–-0.8880
Cut-off value	5.56 ng/ml	38.7 ng/ml	476 U/ml	44.0 ng/ml	107.0 ng/ml
Sensitivity	87.7%	66.2%	96.9%	66.2%	84.6%
Specificity	77.4%	32.3%	93.5%	61.9%	61.3%
Diagnostic accuracy	84.4%	55.2%	95.8%	65.1%	77.1%
Likelihood ratio	3.9	1.0	15.0	1.7	2.2

(b)

	MMP-7	CCL18	KL-6	SP-A	SP-D
AUC	0.9837	0.7767	0.9969	0.8572	0.9673
95% CI	0.9652–-1.0020	0.7140–0.8519	0.9922–1.0020	0.8019–0.9125	0.9442–0.9903
Cut-off value	5.56 ng/ml	38.7 ng/ml	476 U/ml	44.0 ng/ml	107.0 ng/ml
Sensitivity	87.7%	66.2%	96.9%	66.2%	84.6%
Specificity	98.0%	78.2%	100.0%	86.1%	97.0%
Diagnostic accuracy	94.0%	73.5%	98.8%	78.3%	92.2%

Likelihood ratio	44.3	3.0	—	4.8	28.5

ROC: receiver operating characteristic, IPF: idiopathic pulmonary fibrosis, BP: bacterial pneumonia, HC: healthy controls, MMP-7: matrix metalloproteinase-7, CCL18: CC-chemokine ligand 18, KL-6: Krebs von den Lungen-6, SP-A: surfactant protein-A, SP-D: surfactant protein-D, 95% CI: 95% confidence interval

FIGURE S1: ROC curve analysis in serum levels of KL-6, MMP-7 and the combination of serum levels of KL-6 and MMP-7 to distinguish IPF patients from patients with bacterial pneumonia and healthy controls. There was no significant difference between the combination of KL-6 and MMP-7 and either marker alone in the area under the curve of ROC.

FIGURE S2: ROC curve analysis in five biomarkers to distinguish IPF patients from (a) those with bacterial pneumonia and (b) healthy controls.

FIGURE S3: Kaplan-Meier analysis of the IPF patients using biomarker levels above or below the cut-off levels.

FIGURE S4: Martingale residuals plots employed to check for assumptions of the Cox proportional hazards model.

FIGURE S1:

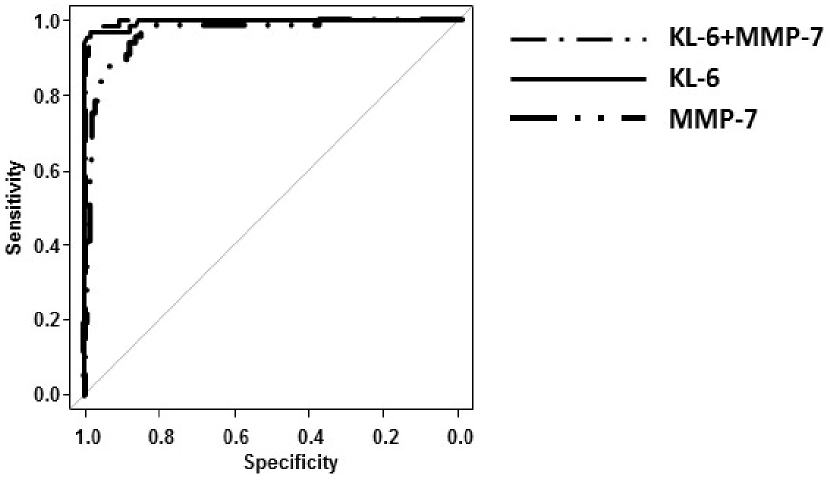
1.0
0.8
0.6
0.4
0.2
0.0
Sensitivity
1.0
0.8
0.6
0.4
0.2
0.0
Specificity
KL-6+MMP-7
KL-6
MMP-7

FIGURE S2:

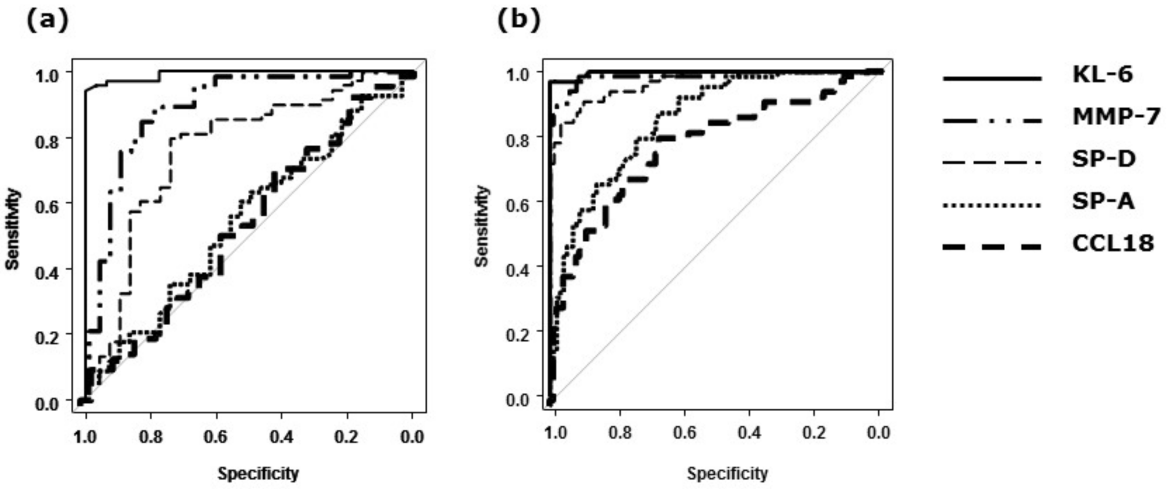
(a)
(b)
Sensitivity
Specificity
1.0
0.8
0.6
0.4
0.2
0.0
KL-6
MMP-7
SP-D
SP-A
CCL18

FIGURE S3:

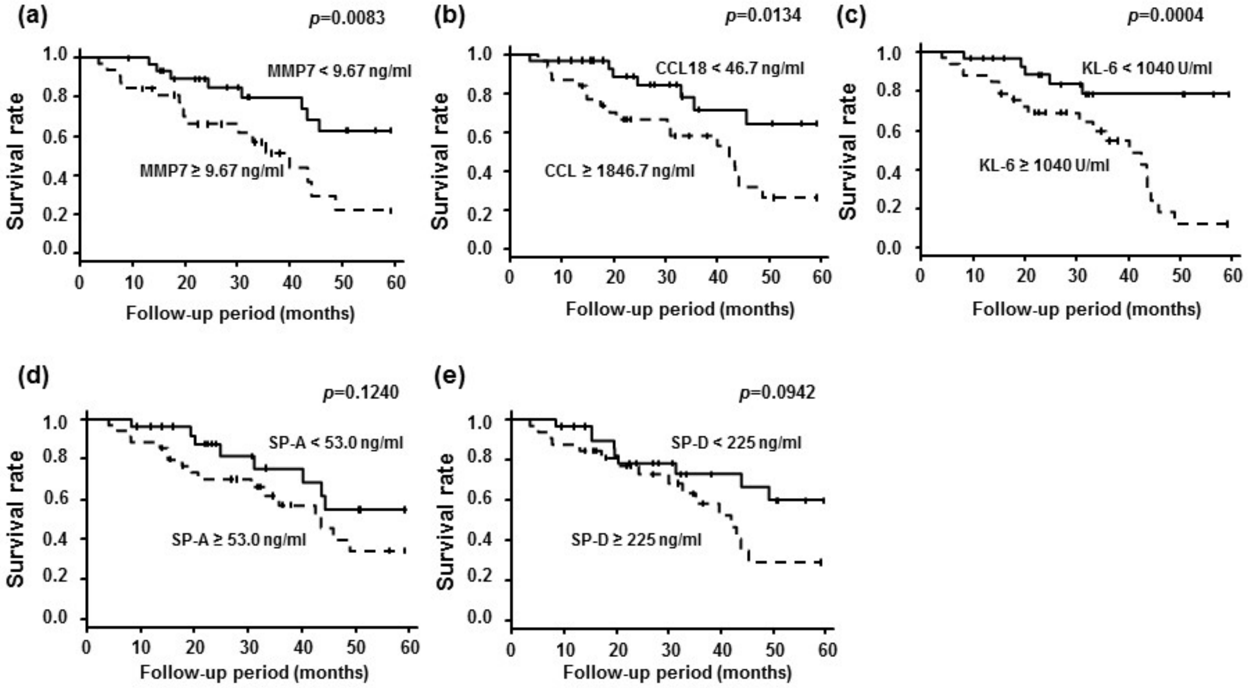
(a)
p=0.0083
Survival rate
MMP7 < 9.67 ng/ml
MMP7 ≥ 9.67 ng/ml
Follow-up period (months)
(b)
p=0.0134
Survival rate
CCL18 < 46.7 ng/ml
CCL ≥ 1846.7 ng/ml
Follow-up period (months)
(c)
p=0.0004
Survival rate
KL-6 < 1040 U/ml
KL-6 ≥ 1040 U/ml
Follow-up period (months)
(d)
p=0.1240
Survival rate
SP-A < 53.0 ng/ml
SP-A ≥ 53.0 ng/ml
Follow-up period (months)
(e)
p=0.0942
Survival rate
SP-D < 225 ng/ml
SP-D ≥ 225 ng/ml
Follow-up period (months)

FIGURE S4:

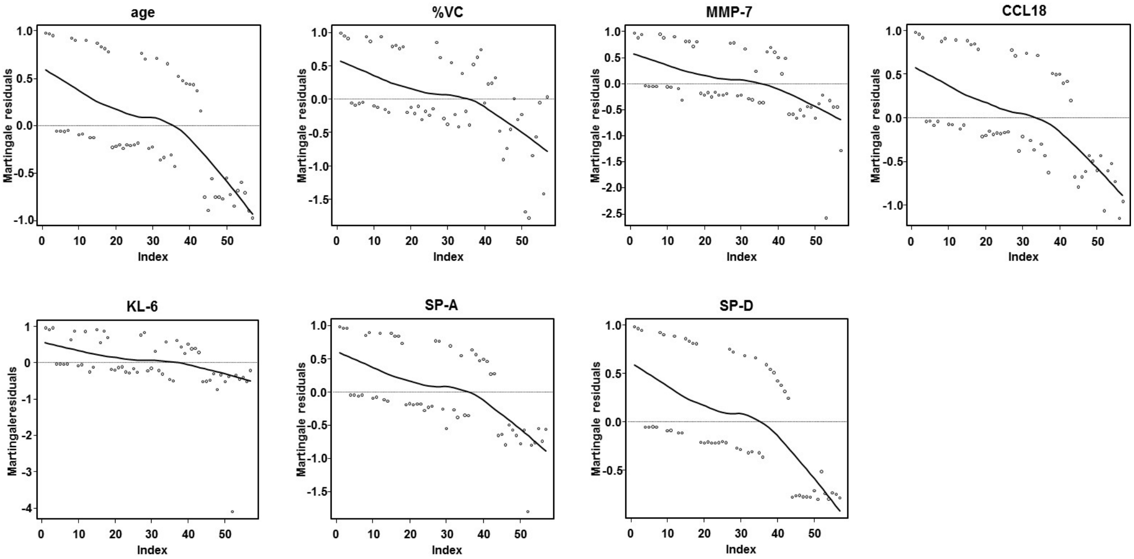
age
%VC
MMP-7
CCL18
KL-6
SP-A
SP-D
Martingale residuals
Martingaleresiduals
Index
